# A live-cell imaging system for visualizing the transport of Marburg virus nucleocapsid-like structures

**DOI:** 10.1186/s12985-019-1267-9

**Published:** 2019-12-19

**Authors:** Yuki Takamatsu, Olga Dolnik, Takeshi Noda, Stephan Becker

**Affiliations:** 1 Institute of Virology, Philipps-University Marburg, Hans-Meerwein-Straße, 35043 Marburg, Germany; 2 Laboratory of Ultrastructural Virology, Institute for Frontier Life and Medical Sciences, Kyoto University, Shogoin-Kawahara-cho 53, Sakyo-ku, Kyoto, 606-8507 Japan; 3 Laboratory of Ultrastructural Virology, Graduate School of Biostudies, Kyoto University, Shogoin-Kawahara-cho 53, Sakyo-ku, Kyoto, 606-8507 Japan; 4 German Center of Infection Research (DZIF), partner site Giessen-Marburg-Langen, Marburg, Germany

**Keywords:** Marburg virus, Nucleocapsid-like structures, Live-cell imaging, Actin polymerization

## Abstract

**Background:**

Live-cell imaging is a powerful tool for visualization of the spatio-temporal dynamics of moving signals in living cells. Although this technique can be utilized to visualize nucleocapsid transport in Marburg virus (MARV)- or Ebola virus-infected cells, the experiments require biosafety level-4 (BSL-4) laboratories, which are restricted to trained and authorized individuals.

**Methods:**

To overcome this limitation, we developed a live-cell imaging system to visualize MARV nucleocapsid-like structures using fluorescence-conjugated viral proteins, which can be conducted outside BSL-4 laboratories.

**Results:**

Our experiments revealed that nucleocapsid-like structures have similar transport characteristics to those of nucleocapsids observed in MARV-infected cells, both of which are mediated by actin polymerization.

**Conclusions:**

We developed a non-infectious live cell imaging system to visualize intracellular transport of MARV nucleocapsid-like structures. This system provides a safe platform to evaluate antiviral drugs that inhibit MARV nucleocapsid transport.

## Background

Marburg virus (MARV), together with Ebola virus (EBOV), belongs to the family *Filoviridae*, and has a roughly 19 kb non-segmented, single-stranded, negative-sense RNA genome. It can cause severe hemorrhagic fever with high fatality rates. MARV epidemics have occasionally been reported in Central Africa, with the largest one, having a 90% fatality rate, being reported in Angola between 2004 and 2005 [1]. MARV infection was also reported outside Central Africa, such as in Germany, South Africa, Russia, and USA [2]. Currently, there are no approved vaccines or antiviral therapeutics available to prevent or treat MARV infection. Therefore, understanding the interplay between viral and host proteins during MARV replication is necessary to establish countermeasures for the diseases. For example, revealing the mechanisms for the assembly and transport of nucleocapsids, which are responsible for the transcription and replication of the viral genome, might contribute to the development of new therapeutic options.

**Fig. 1:**
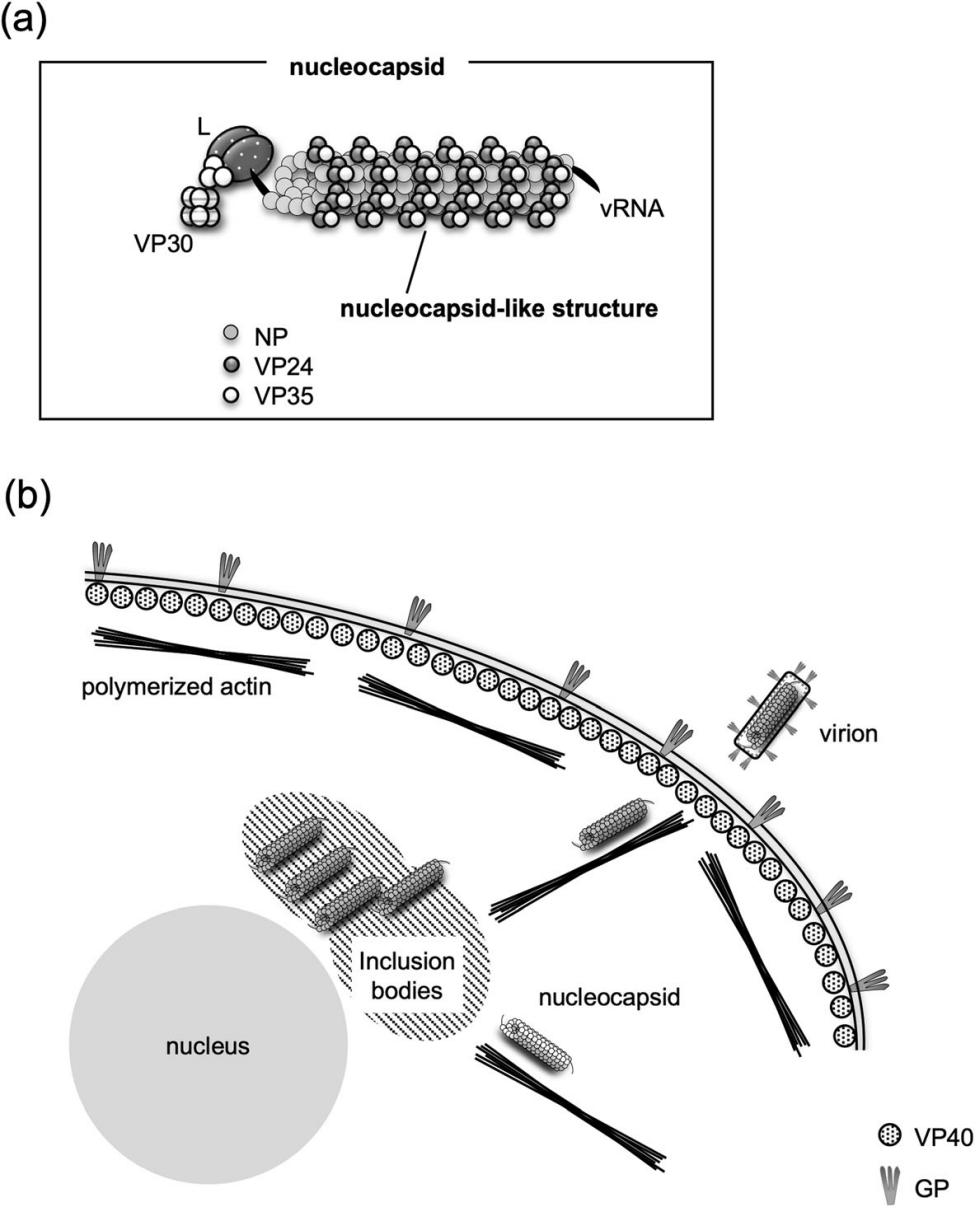
Orientation of MARV nucleocapsid and its transport pathway. **a** The viral genome is encapsidated by NP. Nucleocapsids additionally contain VP24, VP30, VP35, and L. Among them, NP, VP24, and V35 form the core structure of the nucleocapsid called a “nucleocapsid-like structure (NCLS)”. **b** Nucleocapsids are formed in the perinuclear inclusion bodies and are subsequently transported along polymerized actin filaments to the plasma membrane, where budding and release of virions takes place. The nucleocapsid and VP40 protein forms filamentous virions together with GP

The main nucleocapsid protein of MARV is NP, which is responsible for the encapsidation of single-stranded viral genomic RNA [3, 4]. In addition to NP, MARV nucleocapsids also contain the minor matrix protein VP24 and the polymerase cofactor VP35, both of which are essential structural elements that directly interact with NP to build a helical nucleocapsid approximately 900 nm in length and 50 nm in diameter [3, 5, 6]. Furthermore, the viral polymerase L and the transcription factor VP30 are also associated with the nucleocapsid [7, 8]. The core complex of the nucleocapsid, formed by the NP, VP35, and VP24 proteins together with the viral RNA, is defined as a nucleocapsid-like structure (NCLS, Fig. 1a). In MARV- and EBOV-infected cells, immunofluorescence microscopy, as well as live-cell imaging, have been used to visualize and analyze nucleocapsids by fluorescently labeling the nucleocapsid proteins [9–13]. According to these reports, nucleocapsid formation occurs in the perinuclear inclusion bodies, following which they are transported into the cytoplasm and redistributed prior to budding through the cell surface. The velocity of nucleocapsid movement inside cells ranges from 100 nm/s to 500 nm/s in MARV- or EBOV-infection [9, 10]. Application of specific cytoskeleton inhibitors revealed that the transport of MARV nucleocapsids was dependent on actin polymerization. Viral matrix protein VP40, which is a peripheral membrane protein and plays a pivotal role in filamentous virion formation, is essential for the recruitment of nucleocapsids to the cell periphery and for their incorporation into progeny virions [3, 14–16]. The surface glycoprotein GP, which is an integral membrane protein and is responsible for cell entry, forms the filamentous virions together with nucleocapsid and VP40 (Fig. 1b) [11, 17–19].

Live-cell imaging is a powerful tool for visualization of the spatio-temporal dynamics of living organisms. In addition to immunofluorescence microscopy, live-cell imaging microscopy has been utilized to visualize the localization of viral proteins, and interactions between viral and host proteins in various virus-infected cells [11, 20–23]. However, because of its high pathogenicity, MARV must be handled under the highest biosafety conditions [biosafety level 4 (BSL-4)], which complicates and delays research using live-cell imaging [24]. In this study, we developed a safe, live-cell imaging system, following a previously established method for EBOV [25], to visualize MARV nucleocapsid-like structures (NCLSs) in cells expressing viral proteins, outside of BSL-4 laboratories. By using this live-cell imaging system, we were able to analyze interactions between NCLSs and the cellular cytoskeleton, as well as intracellular transport of NCLSs.

## Materials and methods

### Cell culture

Huh-7 (human hepatoma) cells were maintained at 37 °C and 5% CO_2_ in Dulbecco’s Modified Eagle Medium (DMEM, Life Technologies) supplemented with 10% (vol/vol) Fetal bovine serum (FBS, PAN Biotech), 5 mM L-glutamine (Q; Life Technologies), 50 U/mL penicillin, and 50 μg/mL streptomycin (PS; Life Technologies).

### Plasmids and transfection

Plasmids encoding the MARV structural proteins (NP, VP35, VP24, L, VP40 and GP): pCAGGS-NP, pCAGGS-VP35, pCAGGS-VP24, pCAGGS-L, pCAGGS-VP40, and pCAGGS-GP, and a MARV minigenome-expressing plasmid which encodes a *Renilla* luciferase were used [26, 27]. The plasmid pCAGGS-VP30-GFP, coding for the green fluorescent protein-VP30 fusion protein, was produced as previously described [9]. The transfection was performed in 50 μL Opti-MEM without phenol red (Life Technologies) using TranSIT (Mirus) according to the manufacturer’s instructions.

### Live cell imaging microscopy

A total of 2 × 10^4^ Huh-7 cells were seeded onto a μ-Slide 4 well (ibidi) and cultivated in DMEM/PS/Q with 10% FBS. Each well was transfected with the following plasmids, encoding all MARV structural proteins: (250 ng of pCAGGS-NP, 50 ng of pCAGGS-VP35, 125 ng of pCAGGS-VP30-GFP, 50 ng of pCAGGS-VP24, 500 ng of pCAGGS-L, 125 ng of pCAGGS-VP40 and 125 ng of pCAGGS-GP), together with a T7-driven, MARV minigenome-expressing plasmid, which encodes a *Renilla* luciferase, and a T7 polymerase-coding plasmid (pCAGGS-T7) [26, 27]. The inoculum was removed at 1 h post-transfection (p.t.), and 500 μL CO_2_-independent Leibovitz’s medium (Life Technologies) with PS/Q, non-essential amino acid solution, and 20% (vol/vol) FBS were added. Live-cell time-lapse experiments were recorded with a Nikon ECLIPSE TE2000-E using a 63× oil objective or a GE healthcare Delta Vision Elite using a 60× oil objective in biosafety level-2 laboratories.

### Treatment of cells with cytoskeleton-modulating drugs

Cells were treated with 15 μM nocodazole (Sigma), 0.3 μM cytochalasin D (Sigma), or 0.15% dimethyl sulfoxide (DMSO, Sigma), following previous publication [9]. The chemicals were added to the cell culture medium 3 h prior to observation.

### Image processing and analysis

Acquired pictures and movie sequences were processed using the Fiji plugin “TrackMate” [28, 29]. We used LoG detector, which enables detection of the targeted signals to the greatest possible extent. Subsequently, a Simple LAP tracker was used to follow trajectories of the moving signals [28]. The majority of the detected signals were VP30-derived movement without formation of NCLSs, which can be differentiated by shorter trajectory length and random movement pattern [25]. To avoid contamination of the signals derived solely from VP30, we omitted the signals demonstrating trajectory length less than 1 μm and velocity less than 10 nm/s, because they do not conform to the typical transport pattern of NCLSs/nucleocapsids, which is characterized by long-distance directional movement [9, 10, 25]. Previously, we used manual tracking of signals, which primarily demonstrates trajectory length more than 5 μm, track duration over 30 s, and velocity of movement over 100 nm/s [9, 10, 25], although it was not optimized for quantitative analyses.

## Results

### Establishment of a live-cell imaging system for MARV NCLSs transport

**Fig. 2:**
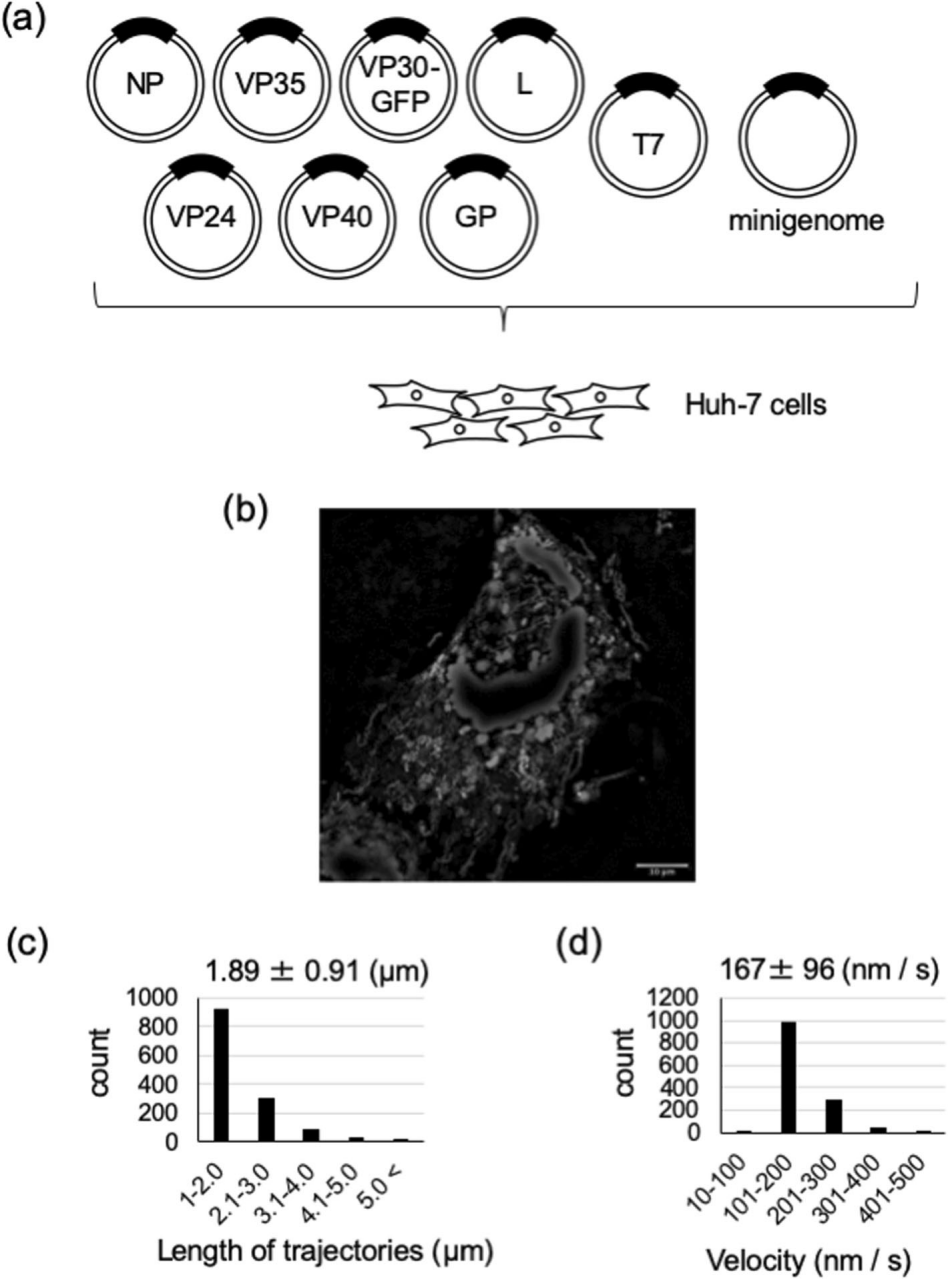
Live-cell imaging system of MARV nucleocapsid-like structures. **a** The experimental setting for detection of NCLS transport. Huh-7 cells were transfected with plasmids encoding NP, L, VP35, VP24, VP40, GP, Marburg virus specific minigenome, T7 polymerase, and VP30-GFP. **b** Plasmid-transfected Huh-7 cells were observed at 18 h p.t. The image shows the maximum-intensity projection of time-lapse images of cells, recorded for 90 s; images were captured every 2 s. **c**, **d** Over 1000 selected signals were analyzed using the Fiji plugin “TrackMate”. **c** The length of the NCLS trajectories was evaluated. The y-axis represents the number of signals in each range (x-axis). The numbers indicate mean ± SD (μm). **d** The velocity of NCLSs transport was evaluated. The y-axis represents the number of signals in each range (x-axis). The numbers indicate mean ± SD (nm/s)

The Marburg virus virus-like particle (VLP) system, which models a complete, single infectious cycle, has been developed and used to analyze viral transcription and replication, as well as the budding processes [26, 27]. In this study, we attempted to visualize MARV NCLSs transport in Huh-7 cells, by using the MARV VLP system and based on the procedure established for EBOV [25]. Cells were transfected with the plasmids encoding the VLP components as illustrated in Fig. 2a. We employed this VLP-based system in all subsequent experiments in this study.

At 18 h p.t., we detected a large number of moving signals with various shapes (Additional file 1: Movie S1). Over 1000 signals in the acquired movie sequences were analyzed. The representing sequence was expressed as the maximum intensity projection, in which the image projected maximum intensity of each time lapse. Among the signals, we focused on those signals showing long-distance and directional transport, which represent NCLSs trajectories [25]. We detected trajectories with lengths ranging from 100 nm to 20 μm, with a mean length of 1.89 ± 0.91 μm (Fig. 2c). The direction of movement of each NCLS also varied, though the cause of this variation remains unclear. The speed of MARV NCLSs transport ranged from 10 nm/s to 500 nm/s, with a mean velocity of 167 ± 96 nm/s (Fig. 2d), which is comparable to the velocity of nucleocapsid transport in MARV-infected cells analyzed in BSL-4 laboratory (106 ± 96 nm/s at plasma membrane, 132 ± 59 nm/s inside filopodia, and 411 ± 87 nm/s in cytoplasm) [9]. In summary, the movement characteristics of NCLSs in our system is similar to those of nucleocapsids in MARV-infected cells.

### Actin polymerization is required for MARV NCLSs transport

**Fig. 3:**
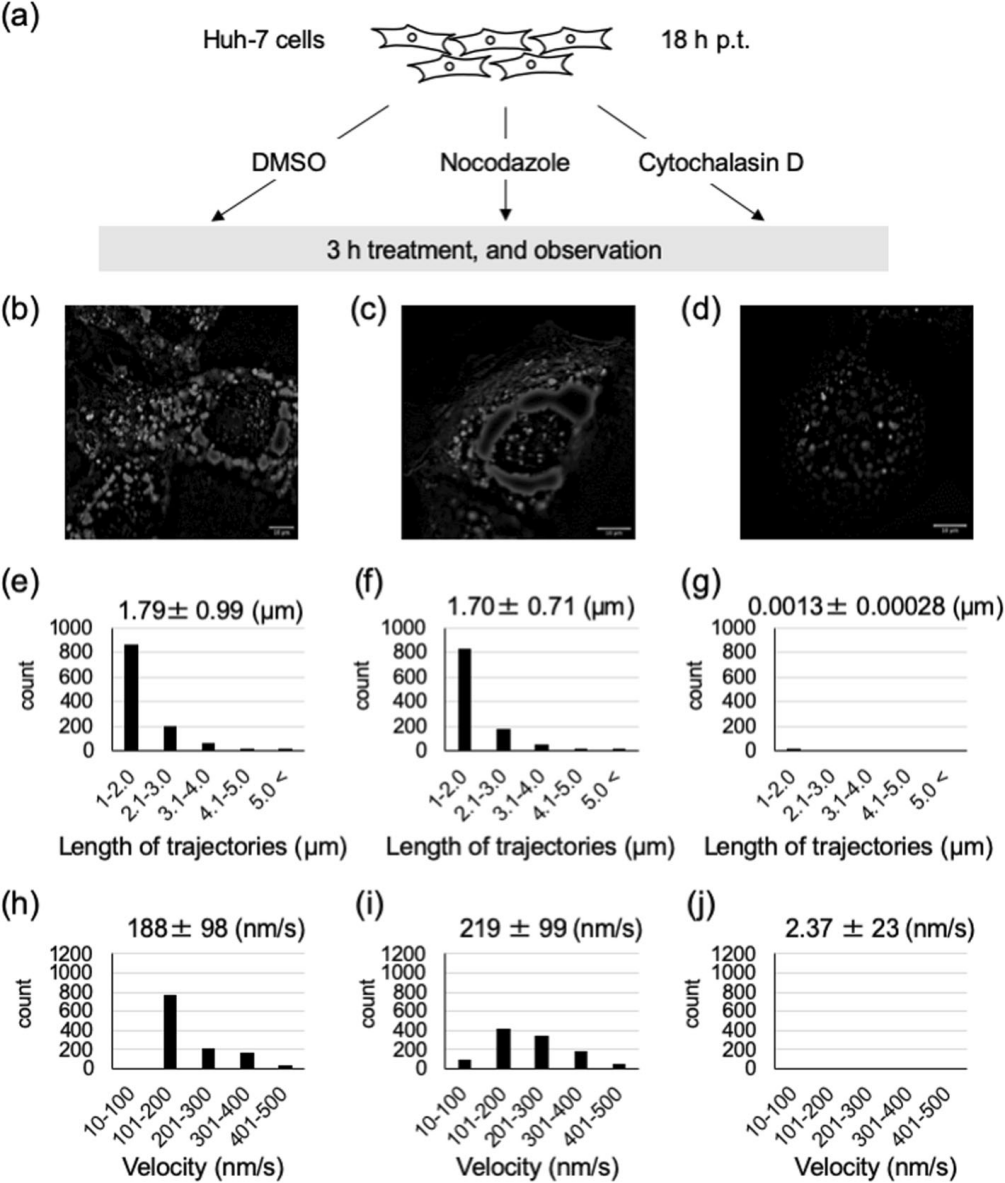
Effect of cytoskeleton-modulating drugs on MARV NCLSs transport. **a** Experimental setting to observe the effects of cytoskeleton modulating drugs on MARV NCLSs transport. Huh-7 cells were transfected with plasmids as described in Fig. 2a, and treated at 15 h p.t. with either 0.15% DMSO (control), 0.15 μM nocodazole, or 0.3 μM cytochalasin D. After 3 h of treatment, observation of the cells began (18 h p.t.). **b**-**d** Time-lapse images were acquired for each of the drug treated cells (b: DMSO, c: Nocodazole, d: Cytochalasin D). The pictures show the maximum-intensity projection of time-lapse images of cells, recorded for 90 s; images were captured every 2 s. **e**-**i** Over 1000 selected signals were analyzed using Fiji plugin “TrackMate”. **e**-**g** The length of the NCLS trajectories was evaluated in each of the drug treated cells (e: DMSO, f: Nocodazole, g: Cytochalasin D). The y-axis represents the number of signals in each range (x-axis). The numbers indicate mean ± SD (μm). **h**-**i** The velocity of NCLSs transport was evaluated in each of the drug treated cells (h: DMSO, i: Nocodazole, **j**: Cytochalasin D). The y-axis represents the number of signals in each range (x-axis). The numbers indicate mean ± SD (nm/s)

In MARV-infected cells, the microtubule depolymerizing drug nocodazole does not alter the movement of nucleocapsids, whereas the actin depolymerizing drug cytochalasin D arrests it [9]. To confirm the relevance of the live-cell imaging system we developed in this study, we analyzed NCLSs movement after treatment with cytoskeletal modulating drugs. Huh-7 cells were transfected with plasmids as described in Fig. 2a. The culture medium was replaced at 15 h p.t. with Leibovitz’s medium containing either 0.15% DMSO (control), 0.15 M nocodazole, or 0.3 μM cytochalasin D (Fig. 3a-d, Additional file 2: Movie S2, Additional file 3: Movie S3, Additional file 4: Movie S4) [9]. After incubating the cells with cytoskeletal modulating drugs for 3 h, time-lapse images were acquired. Nocodazole treatment did not alter the trajectory length of NCLSs transport in comparison to the control, whereas cytochalasin D treatment induced immediate cessation of long-distance transport (Fig. 3e-g). The mean velocity of NCLSs transport in the control or nocodazole-treated cells was 188 ± 98 nm/s and 219 ± 99 nm/s, respectively (Fig. 3h). The mean velocity of nucleocapsid transport in MARV-infected cells treated with DMSO and nocodazole was 379 ± 61 nm/s and 334 ± 55 nm/s, respectively [9]. A reduction in mean velocity is derived from the difference in the inclusion criteria for analysis, which was described in the Materials and Methods. Briefly, the trajectories of long-distance signals in the cytoplasmic region were analyzed in the previous reports [9, 25], whereas we applied software-based quantitative analysis, including the signals in the cytoplasm, plasma membrane, and inside filopodia, in the current study. On the other hand, only a few non-specific NCLSs movements were detectable in cytochalasin D-treated cells, with a mean velocity of 2.73 ± 23 nm/s (Fig. 3i-j). These results confirmed that NCLSs transport is dependent on actin polymerization, as well as the suitability of our assay to test candidate drugs which target intracellular transport of nucleocapsid.

## Discussion

In the present study, we developed a live-cell imaging system for cells expressing MARV proteins, which can be safely used without BSL-4 laboratories. Furthermore, we demonstrated the relevance of our system as a substitute for the analysis of nucleocapsids transport in MARV-infected cells.

We previously developed a live-cell imaging system visualizing intracellular transport of NCLSs in EBOV proteins-expressing cells [25]. According to this, the transport characteristics of EBOV NCLSs, velocity and mediated by actin polymerization, are similar to those of nucleocapsids observed in EBOV-infected cells [10, 25]. In the previous reports, we manually measured and calculated the trajectory length and velocity of nucleocapsids and NCLSs transport [9, 10, 25]. To improve quantity of analysis for NCLSs transport, here we applied automatic detection of moving signals using Fiji plugin TrackMate [28]. By adopting selection criteria, we collected sufficient moving signals except for Brownian like random movement. The calculated velocity of moving NCLSs is similar in both methods.

Currently, cellular factors involved in the nucleocapsid transport have not been fully understood. Although there are several actin-dependent motor proteins such as myosins, and actin-associated proteins necessary for polymerization, such as Arp2/3 and N-WASP, only Arp2/3 was known to be directly associated with Ebola virus nucleocapsid transport [10]. The combined approach of gene silencing and inhibitor screening using the system we developed might represent a powerful tool to identify the key host factors for the intracellular transport of MARV nucleocapsids. Moreover, it is noteworthy that the technical approach developed here might be applicable to study the nucleocapsid transport of other mononegaviruses, as well as to characterize antivirals inhibiting nucleocapsid transport.

## Conclusion

We developed a live-cell imaging system for cells expressing MARV proteins, which can be safely used without BSL-4 laboratories, and demonstrated the relevance of our system as a substitute for the analysis of nucleocapsids transport in MARV-infected cells. Our developed live-imaging system might contribute to study the nucleocapsid transport of other mononegaviruses, as well as to characterize antivirals inhibiting nucleocapsid transport.

## Supplementary information

**Additional file 1 **: **Movie S1.** Huh-7 cells were transfected with plasmids encoding NP, L, VP35, VP24, VP40, GP, Marburg virus specific minigenome, T7 polymerase, and VP30-GFP and observed at 18 h p.t. Time-lapse images of cells, recorded for 90 s; images were captured every 2 s.

**Additional file 2 **: **Movie S2.** Huh-7 cells were transfected with plasmids encoding NP, L, VP35, VP24, VP40, GP, Marburg virus specific minigenome, T7 polymerase, and VP30-GFP. The cells were treated at 15 h p.t. with 0.15% DMSO for 3 h. Time-lapse images of cells were recorded for 90 s; images were captured every 2 s.

**Additional file 3 **: **Movie S3.** Huh-7 cells were transfected with plasmids encoding NP, L, VP35, VP24, VP40, GP, Marburg virus specific minigenome, T7 polymerase, and VP30-GFP. The cells were treated at 15 h p.t. with 0.15 μM nocodazole for 3 h. Time-lapse images of cells were recorded for 90 s; images were captured every 2 s.

**Additional file 4 **: **Movie S4.** Huh-7 cells were transfected with plasmids encoding NP, L, VP35, VP24, VP40, GP, Marburg virus specific minigenome, T7 polymerase, and VP30-GFP. The cells were treated at 15 h p.t. with 0.3 μM cytochalasin D for 3 h. Time-lapse images of cells were recorded for 90 s; images were captured every 2 s.

## Data Availability

The datasets used and/or analyzed in the current study are available from the corresponding author upon reasonable request.

## Abbreviations

BSL: Biosafety level
DMEM: Dulbecco’s Modified Eagle Medium
DMSO: Dimethyl sulfoxide
EBOV: Ebola virus
FBS: Fetal bovine serum
MARV: Marburg virus
NCLS: Nucleocapsid-like structure
NP: Nucleoprotein
p.t: post-transfection
PS: Penicillin and streptomycin
Q: L-glutamine
SD: Standard deviation
VLP: Virus-like particle
